# Efficient Demyristoylase Activity of SIRT2 Revealed by Kinetic and Structural Studies

**DOI:** 10.1038/srep08529

**Published:** 2015-02-23

**Authors:** Yan-Bin Teng, Hui Jing, Pornpun Aramsangtienchai, Bin He, Saba Khan, Jing Hu, Hening Lin, Quan Hao

**Affiliations:** 1 Department of Biomedical Sciences, Cornell University, Ithaca, NY 14853, USA; 2 Department of Chemistry and Chemical Biology, Cornell University, Ithaca, NY 14853, USA; 3 Department of Physiology, University of Hong Kong, Hong Kong, China

## Abstract

Sirtuins are a class of enzymes originally identified as nicotinamide adenine dinucleotide (NAD)-dependent protein lysine deacetylases. Among the seven mammalian sirtuins, SIRT1-7, only SIRT1-3 possess efficient deacetylase activity *in vitro*, whereas SIRT4-7 possess very weak *in vitro* deacetylase activity. Several sirtuins that exhibit weak deacetylase activity have recently been shown to possess more efficient activity for the removal other acyl lysine modifications, such as succinyl lysine and palmitoyl lysine. Here, we demonstrate that even the well-known deacetylase SIRT2 possesses efficient activity for the removal of long-chain fatty acyl groups. The catalytic efficiency (*k_cat_/K_m_*) for the removal of a myristoyl group is slightly higher than that for the removal of an acetyl group. The crystal structure of SIRT2 in complex with a thiomyristoyl peptide reveals that SIRT2 possesses a large hydrophobic pocket that can accommodate the myristoyl group. Comparison of the SIRT2 acyl pocket to those of SIRT1, SIRT3, and SIRT6 reveals that the acyl pockets of SIRT1-3 are highly similar, and to a lesser degree, similar to that of SIRT6. The efficient *in vitro* demyristoylase activity of SIRT2 suggests that this activity may be physiologically relevant and warrants future investigative studies.

Sirtuins, which are represented by the founding member yeast SIR2 (silencing information regulator 2), are a class of enzymes that possess Nicotinamide Adenine Dinucleotide (NAD^+^)-dependent protein lysine deacetylase activity and were originally discovered in the aging and epigenetic fields^1^. Sirtuins regulate numerous biological pathways by deacetylating various substrate proteins, including histones, transcription factors, and metabolic enzymes^2^,^3^,^4^. Mammals possess seven sirtuins, SIRT1-7^5^, which share a conserved catalytic core domain but differ in their N- and C- termini. SIRT1, 6 and 7 are located in the nucleus. SIRT3, 4 and 5 are located in mitochondria and are involved in energy metabolism and responses to oxidative stress. SIRT2 is the only primarily cytoplasmic isoform^6^; however, it has also been observed in the nucleus.

Among the seven sirtuins, only SIRT1-3 (belonging to class I sirtuins) possess efficient deacetylase activity *in vitro*^7^, whereas SIRT4–7 exhibit little or undetectable deacetylation activity. Recently, we have demonstrated that SIRT5, which is a class III mitochondrial sirtuin that exhibits weak deacetylase activity, can efficiently remove negatively charged acyl groups, such as succinyl and malonyl groups, due to the presence of an Arg and a Tyr in the SIRT5 active site^8^. Although protein lysine succinylation and malonylation have not been previously known as common protein posttranslational modifications, it is currently established that these modifications are abundant in both bacteria and mammals^9^,^10^,^11^,^12^. We have also demonstrated that SIRT6, which is a class IV sirtuin that exhibits weak deacetylase activity, can remove long-chain fatty acyl groups more efficiently^13^. These studies have established that sirtuins are NAD-dependent protein lysine deacylases and that different sirtuins may exhibit different acyl group preferences.

The defatty-acylase activity of sirtuins may be more widespread. A malaria parasite SIR2, PfSIR2A, was also shown to possess a similar activity toward long-chain fatty acyl groups^14^. Perhaps more surprisingly, we and others have recently found that even SIRT1-3, which exhibit efficient deacetylase activities, can also remove long-chain fatty acyl groups^15^,^16^. However, the efficiency and physiological relevance of this activity of SIRT1-3 are not known. Here, we sought to determine the efficiency of the defatty-acylase activity compared to the deacetylase activity of SIRT2 and the structural basis for the recognition of long-chain fatty acyl groups by SIRT2. Our study reveals that SIRT2 prefers myristoyl lysine as a substrate slightly over acetyl lysine. A crystal structure of SIRT2 in complex with a thiomyristoyl peptide reveals a hydrophobic pocket responsible for the recognition of long-chain fatty acyl groups. Our studies demonstrate that the defatty-acylase activity of SIRT2 is highly efficient and provide compelling rationale for future studies on the physiological relevance of this activity.

## Results

### SIRT2 can remove long-chain fatty acyl groups from different peptide sequences

**Figure 1:**
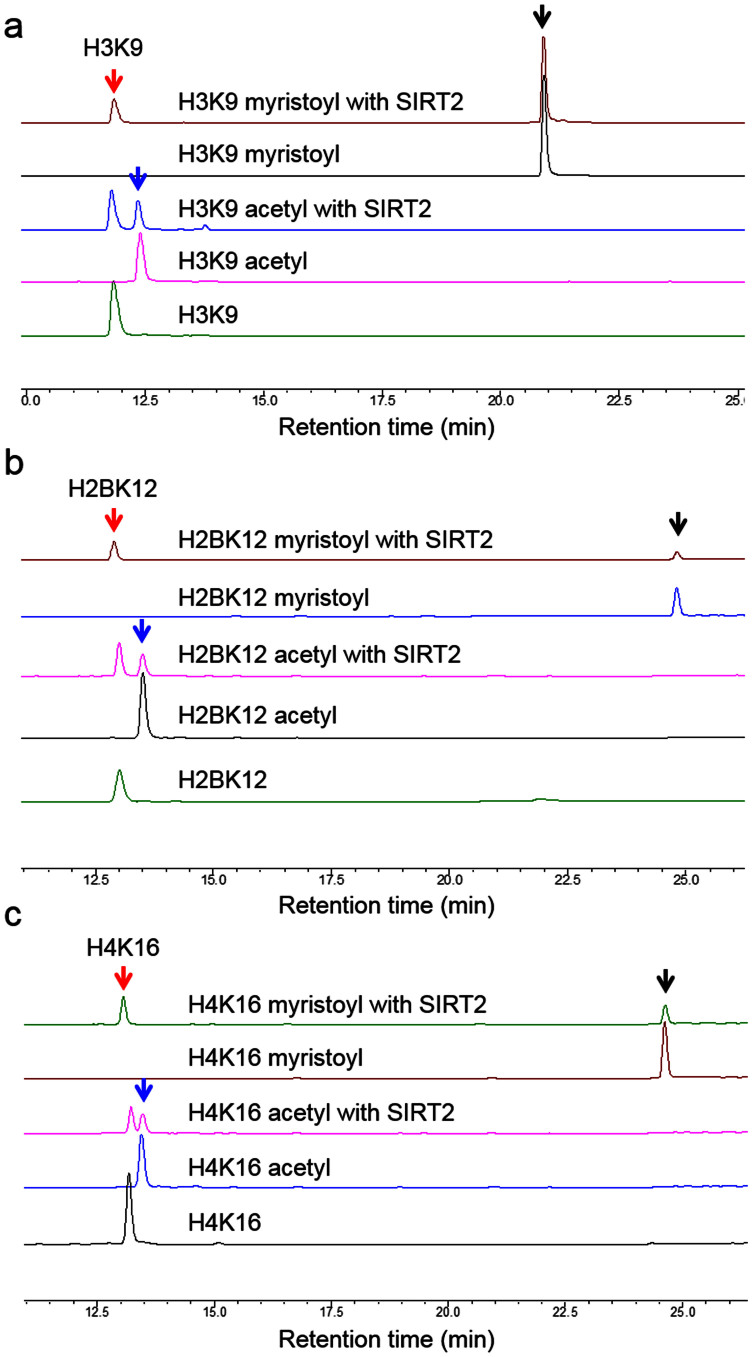
Overlaid HPLC traces showing that SIRT2 could hydrolyze acetyl and myristoyl groups from different peptide sequences, H3K9 (a), H2BK12 (b) and H4K16 (c). The unmodified, acetylated and myristoylated peptides are indicated by the red, blue and black arrows, respectively.

Previous studies demonstrating the defatty-acylase activity of SIRT1-3 only used one peptide sequence^15^,^16^. We first evaluated whether SIRT2 can demyristoylate different peptide sequences. In addition to histone H3 Lys9 (H3K9)^17^ (Figure 1a) peptides, we synthesized histone H2B Lysine12 (H2BK12) and histone H4 Lysine16 (H4K16) peptides bearing acetyl and myristoyl groups. The SIRT2 protein used herein consists of residues 38–356 of the full-length human SIRT2. SIRT2 was able to remove both acetyl and myristoyl groups from H2BK12 (Figure 1b) and H4K16 (Figure 1c) peptides without a strong sequence bias, suggesting that SIRT2 may exert demyristoylase activity toward a wide range of substrates. Compared with the deacetylase activity, the demyristoylase activity of SIRT2 requires a longer time to reach a similar conversation rate (Figure 1), suggesting that the *k_cat_* of demyristoylation is lower than that of deacetylation.

### The demyristoylase activity of SIRT2 is more efficient than its deacetylase activity

To quantitatively compare the activity of SIRT2 toward acetyl and long-chain fatty acyl peptides, we performed kinetics studies using acetyl and myristoyl H3K9 peptides, respectively. As shown in Table 1, although the *k*_cat_ for deacetylation (0.275 s^−1^) was 15-fold higher than that for demyristoylation (0.018 s^−1^), the *K*_m_ for deacetylation (19.00 μM) was approximately 80-fold higher than that for demyristoylation (0.24 μM). Therefore, *k*_cat_/*K*_m_ for demyristoylation (74,000s^−1^M^−1^) was approximately 5-fold higher than that for deacetylation (14,500 s^−1^M^−1^).

### Crystal structure of SIRT2 in complex with a thiomyristoyl peptide, BHJH-TM1

**Figure 2:**
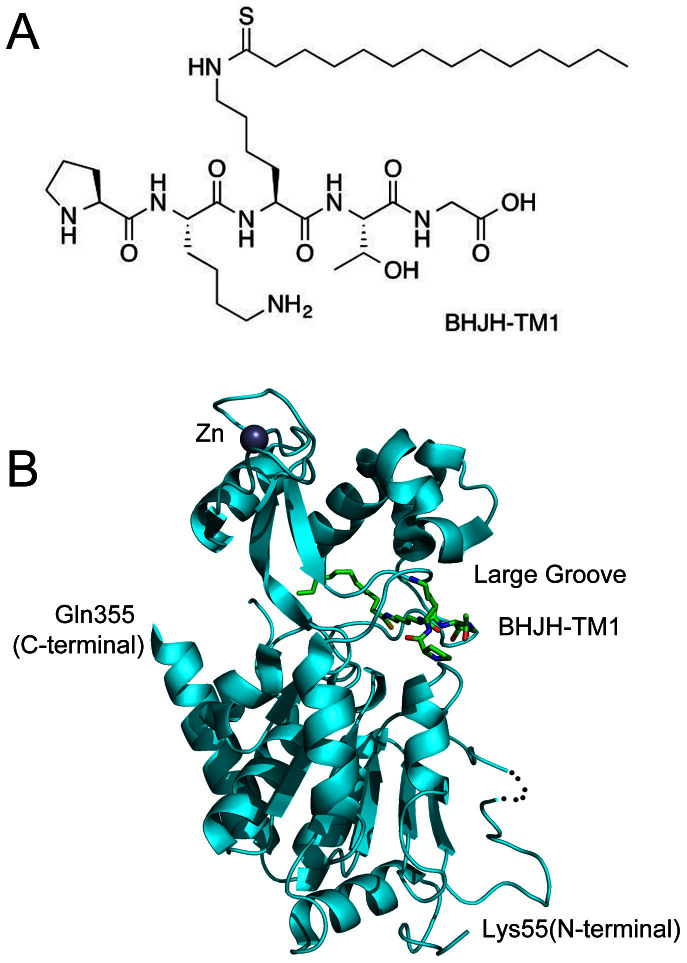
Overall Structure of SIRT2 in complex with a thiomyristoyl peptide, BHJH-TM1. (A) Structure of the BHJH-TM1. (B) The overall structure of SIRT2/BHJH-TM1 complex.

To further understand the defatty-acylase activity of SIRT2, we sought to obtain an X-ray crystal structure of SIRT2 in complex with a myristoyl peptide substrate or analog. Upon screening of crystallization conditions, we obtained crystals of SIRT2 in complex with a thiomyristoyl peptide, referred to as BHJH-TM1 (Figure 2A). BHJH-TM1 was synthesized as a mechanism-based inhibitor of SIRT1-3 and SIRT6^16^. The crystal structure of SIRT2/BHJH-TM1 was refined to a resolution of 2.1 Å, with two subunits in the asymmetric unit. Most of the residues (residues 55–355 from chain A and residues 54–355 from chain B) fit well in the observed electron density, except for the loop regions consisting of residues 293–305 in both subunits, residues 139–140 in chain A and residues 98–101 in chain B.

As observed in the crystal structures of apo SIRT2 and other sirtuins, SIRT2/BHJH-TM1 consists of two distinct domains: a Rossmann fold domain and a zinc-binding domain. The BHJH-TM1 peptide inserts into a large groove between the two domains (Figure 2B).

### BHJH-TM1 binding site of SIRT2

**Figure 3:**
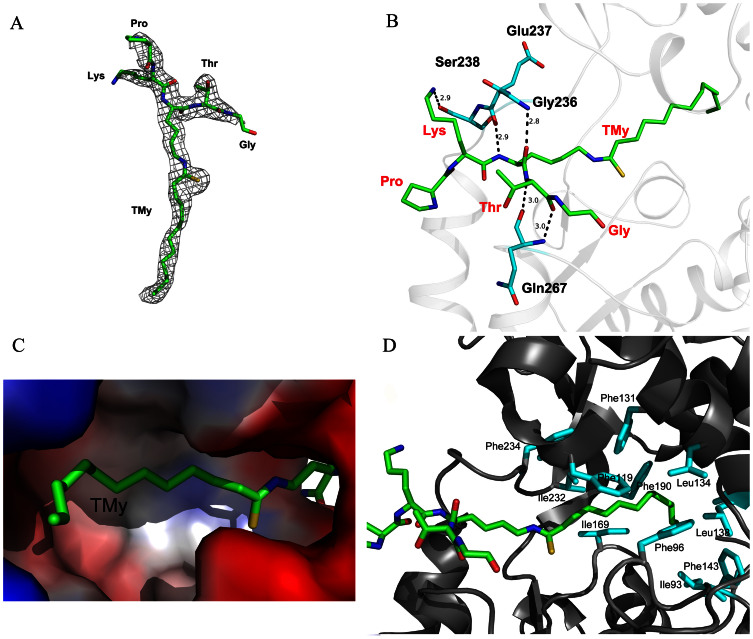
The active site of SIRT2/BHJH-TM1. (A) Electron density map of the BHJH-TM1. The 2Fo-Fc map is colored in grey and contoured at 1.5 σ. (B) The hydrogen bonding interactions between SIRT2 and BHJH-TM1. (C) Surface representation of myristoyl-binding pocket. The surface potential is displayed as a color gradient from red (negative) to blue (positive). (D) Hydrophobic residues (colored cyan) of SIRT2 that are involved in binding the thiomyristoyl group of BHJH-TM1.

The BHJH-TM1 peptide (PKK(TMy)TG) consists of five amino acid residues (Figure 2A) with a thiomyristoyl group attached to the central lysine residue. The BHJH-TM1 peptide fits well into a long stretch of electron density observed between the N-terminal Rossmann fold domain and C-terminal zinc-binding domain of SIRT2 (Figure 3A). The binding mode of the BHJH-TM1 peptide to SIRT2 is similar to that observed for other sirtuins (e.g., *Archaeoglobus fulgidus* SIR2^18^ and human SIRT5^8^) and predominantly involves the following hydrogen bonds (Figure 3B): the main chain NH and CO of the thiomyristoyl lysine form hydrogen bonds with the main chain CO of Glu237 and the main chain NH of Gly236, respectively; the main chain NH and CO of threonine form hydrogen bonds with the main chain CO and NH of Gln267, respectively; and the Nε of the free lysine side chain forms a hydrogen bond with the side chain OH of Ser238 (Figure 3B). Compared to the apo SIRT2 structure, all four residues involved in these hydrogen bonds lie far from the BHJH-TM1 molecule in the apo SIRT2 structure. This finding indicates that the entrance of the binding site is more open prior to substrate binding, facilitating substrate access.

In both active sites of the two subunits, the BHJH-TM1 molecule is finely complementary to the site in terms of both shape and charge (Figure 3C). The thiomyristoyl group of BHJH-TM1 is accommodated by a hydrophobic pocket formed by several hydrophobic residues of SIRT2, including Ile93, Phe96, Phe119, Phe131, Leu134, Leu138, Phe143, Ile169, Phe190, Ile232, and Phe234 (Figure 3D). The extensive hydrophobic interactions between the thiomyristoyl group and SIRT2 may account for the much lower *K_m_* value of SIRT2 toward the myristoyl lysine peptide (Table 1).

### Comparison of the apo SIRT2 and SIRT2/BHJH-TM1 structures

**Figure 4:**
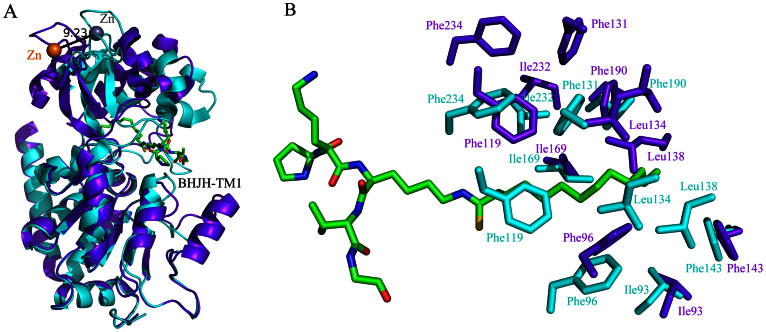
Substrate binding triggers the formation of a closed state of SIRT2. (A) Alignment of SIRT2 apo structure (purple) and SIRT2/BHJH-TM1 structure (cyan). The apo structure is more open. Upon substrate binding, the zinc binding domain (top) moves toward the Rossmann fold domain to bind the substrate analog BHJH-TM1. (B) Detailed view of the residues forming the acyl pocket also reveals the movement of the residues from the top zinc-binding domain towards the lower Rossmann fold domain. The substrate analog BHJH-TM1 is shown in green stick representation and for clarity, the side chains are not shown except for the thiomyristoyl lysine.

Comparison of the overall structures of SIRT2/BHJH-TM1 and apo SIRT2 (PDB ID: 3ZGO) indicates that the zinc-binding domain moves approximately 10 Å toward the groove, closing the binding site in the SIRT2/BHJH-TM1 structure (Figure 4A). A similar conformational change was also observed in SIRT1^19^. Comparison of the active sites of SIRT2/BHJH-TM1 and apo SIRT2 (Figure 4B) reveals that the hydrophobic residues from the Rossmann fold domain that form the myristoyl pocket (e.g., Ile93, Phe143, and Ile169; Figure 4B) superpose relatively well, whereas the hydrophobic residues from the zinc-binding domain (e.g., Phe131, Leu138, and Phe234) move approximately 3–4 Å between the apo SIRT2 structure and the SIRT2/BHJH-TM1 structure (Figure 4B). This conformational change reshapes the binding site, which may better accommodate the thiomyristoyl group.

### Comparison of the SIRT2/BHJH-TM1 structure with the structures of SIRT3 and SIRT6

**Figure 5:**
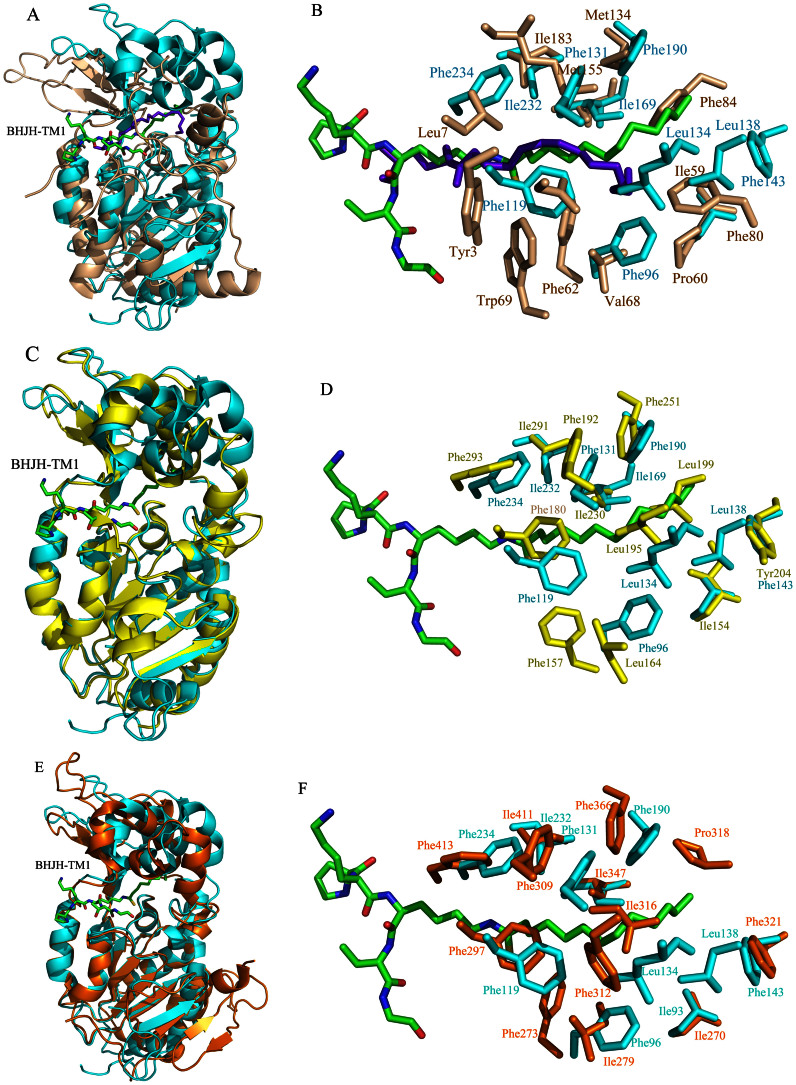
Structural comparison between SIRT2 and SIRT6 (PDB 3ZG6)/SIRT3 (PDB 3GLT). (A) Overall structural alignment of SIRT2 (cyan) and SIRT6 (wheat). (B) Detailed view of the acyl pockets of SIRT2 and SIRT6. The myristoyl group in SIRT6 structure is shown in purple, the thiomyristoyl group in SIRT2 structure is shown in green. Residues from SIRT6 are shown in wheat, while residues from SIRT2 are shown in cyan. (C) Overall structural alignment of SIRT2 (cyan) and SIRT3 (yellow). (D) Detailed view of the acyl pockets of SIRT2 and SIRT3. The thiomyristoyl group in SIRT2 structure is shown in green. Residues from SIRT3 are shown in yellow, while residues from SIRT2 are shown in cyan. (E) Overall structural alignment of SIRT2 (cyan) and SIRT1 (brown). (F) Detailed view of the acyl pockets of SIRT2 and SIRT1. The thiomyristoyl group in SIRT2 structure is shown in green. Residues from SIRT1 are shown in brown, while residues from SIRT2 are shown in cyan.

We have previously reported that SIRT6 prefers to hydrolyze long-chain fatty acyl groups from protein lysine residues *in vitro*. As observed in SIRT2, SIRT6 also possesses a large hydrophobic pocket that accommodates the long-chain fatty acyl groups^13^. We superposed the structures of SIRT2 and SIRT6 (PDB ID: 3ZG6) to compare their acyl pockets. Although the Rossmann fold domains and the bound acyl lysine peptides superpose relatively well, the structures of the SIRT2 and SIRT6 zinc-binding domains are quite different (Figure 5A). The hydrophobic residues that form the acyl pockets are also very different (Figure 5B). For example, Met134 in SIRT6 corresponds to the position of Phe190 in SIRT2, and Asn2 and Trp69 in SIRT6 occupy the corresponding position of Phe119 in SIRT2. Most strikingly, Phe84 in SIRT6 occupies a position that would sterically clash with the more extended thiomyristoyl group (green color, Figure 5B) in the SIRT2 structure. Phe84 in SIRT6 forces the myristoyl group in complex with SIRT6 to adopt a more bent conformation (purple color, Figure 5B). Despite these differences (in terms of shape and residues involved) between the two acyl pockets in SIRT2 and SIRT6, these pockets lie at approximately similar locations in both proteins to accommodate the long-chain fatty acyl groups. Because hydrophobic side chains are critical for the formation of an acyl pocket, it is perhaps not surprising that the residues that form the acyl pockets in SIRT2 and SIRT6 are different.

Comparison of the overall structures of SIRT2/BHJH-TM1 with SIRT3 (PDB ID: 3GLT)^20^ or SIRT1 (PDB ID: 4KXQ)^19^ reveals that the structures are highly similar (Figures 5C and 5E). The acyl pockets are also highly similar (Figures 5D and 5F). Several of the hydrophobic residues that form the acyl pocket are identical. For example, Phe293, Ile291, Phe192 and Phe251 in SIRT3 superpose well with Phe234, Ile232, Phe131, and Phe190 of SIRT2, respectively (Figure 5D). The most notable difference between the SIRT3 (or SIRT1) acyl pocket and the SIRT2 acyl pocket is that Leu199 of SIRT3 (or Ile316 of SIRT1) lies much closer to the thiomyristoyl group than the corresponding Leu138 of SIRT2, which may result in steric clashes (Figures 5D and 5F). This potential steric clash may force the myristoyl group to turn toward or into the empty space beneath Leu164 of SIRT3 (or Ile279 of SIRT1), creating a bent myristoyl conformation that is similar to that observed in SIRT6, as shown in Figure 5B. Given that SIRT3 and SIRT1 can also hydrolyze long-chain fatty acyl groups, this structural comparison provides a reasonable model for the recognition of long-chain fatty acyl groups by SIRT3 and SIRT1.

## Discussion

We have demonstrated that SIRT2 is an efficient demyristoylase *in vitro*. The catalytic efficiency of SIRT2 for the hydrolysis of myristoyl lysine is slightly higher than that for deacetylation. This increased catalytic efficiency originates primarily from the decreased *K_m_* values. In contrast, the *k_cat_* value for demyristoylation is lower than that for deacetylation. The crystal structure of SIRT2 in complex with a thiomyristoyl peptide, BHJH-TM1, reveals a larger hydrophobic pocket that is responsible for binding the long-chain fatty acyl groups. The hydrophobic acyl pocket of SIRT2 resembles that of SIRT6, which is a sirtuin that has been previously demonstrated to possess efficient defatty-acylase activity, although the two acyl pockets differ in certain aspects. In addition, the hydrophobic acyl pocket of SIRT2 is highly similar to that of SIRT1 and SIRT3, thus providing a reasonable model to account for the ability of SIRT1 and SIRT3 to remove long-chain fatty acyl groups. Moreover, SIRT1, SIRT2 and SIRT3 can remove lipoic acid based on assays utilizing [^32^P]NAD^+^^21^. For other long-chain fatty acyl substrates, such as hexanoyl, octanoyl, and decanoyl groups, SIRT2 also exhibits defatty-acylase activity; however, the defatty-acylase activity toward palmitoyl groups is not efficient, which may result from the limited depth of the active site.

Sirtuins are classified into four different classes based on sequence similarity^5^. SIRT1-3, which were previously thought be primarily deacetylases, all belong to class I. SIRT4, which does not exhibit efficient activity *in vitro*, belongs to class II. The desuccinylase/demalonylase/deglutarylase SIRT5 belongs to class III. SIRT6 (preferentially a defatty-acylase *in vitro*) and SIRT7 both belong to be class IV. Our kinetic and structural studies on SIRT2 suggest that the defatty-acylase activity of SIRT2 (and likely that of SIRT1 and SIRT3) is equally efficient, if not more efficient, than the deacetylase activity. Thus, class I sirtuins have likely evolved to recognize both short and long-chain fatty acyl groups. Class IV sirtuins, particularly SIRT6, exhibit a preference for long-chain fatty acyl groups, at least *in vitro*. However, several studies suggest that the deacetylase activity is important *in vivo*. Class III sirtuins preferentially recognize negatively charged acyl groups. Human SIRT5 is specific for negatively charged acyl groups, such as malonyl, succinyl, and glutaryl groups. However, certain class III members, such as *Escherichia coli* CobB, may be multifunctional and can remove different acyl groups (such as succinyl and acetyl) with similar efficiency^22^. Thus, it appears that many sirtuins can exhibit promiscuous activities toward different acyl lysine modifications.

Our kinetic and structural analyses of SIRT2 suggest that the defatty-acylase activity may be physiologically relevant. A recent study shows that SIRT2 can regulate protein lysine fatty-acylation in cells^23^. The physiological relevance of this defatty-acylase activity is particularly interesting for several reasons. First, this activity is primarily localized in the cytosol (in contrast to SIRT1, which is primarily nuclear, and SIRT3, which is primarily mitochondrial)^7^. Most known protein fatty acyl modifications, such as N-terminal glycine myristoylation and cysteine palmitoylation, occur in the cytosol or the cytosolic face of the ER and Golgi^24^. Therefore, it is reasonable to speculate that numerous lysine fatty acylation events will also occur in the cytosol and be regulated by cytosolic SIRT2. Second, in contrast to SIRT1, for which numerous deacetylation substrates have been identified, relatively few deacetylation substrates of SIRT2 have been reported. These SIRT1 substrates include α-tubulin^25^, histones H3 and H4^26^,^27^,^28^, lactate dehydrogenase A^29^, BubR1^30^, and HIF-1α^31^. Consistent with this observation, proteomic studies on both SIRT1 and SIRT3 have reported the identification of hundreds of substrates^32^,^33^,^34^, in contrast to the lack of similar studies for SIRT2. The limited number of identified deacetylation substrates for SIRT2 may reflect an as-of-yet unidentified biochemical function. Efforts to identify the defatty-acylation substrate of SIRT2 are currently ongoing.

## Methods

### Materials

Acetyl and myristoyl peptide substrates used in the SIRT2 assay were synthesized as previously reported. The thiomyriostyl peptide BHJH-TM1 was synthesized as a mechanism-based inhibitors for SIRT1-3 and SIRT6 as recently reported^16^.

### Overexpression and Purification of SIRT2

The *Sirt2* gene from *Homo.Sapiens* was cloned into a pET28b(+) (Novagen) vector containing an N-terminal hexahistidine and sumo tag. SIRT2 was overexpressed in *E. coli* BL21 (DE3) grown in Luria-Bertani (LB) media. The cells were grown at 37°C to an OD_600_ of ~0.6 and induced with 0.2 mM isopropyl 1-β-D-galactopyranoside (IPTG) for 18–24 h at 18°C. Cell pellets from 2 L of culture were lysed by sonication on ice in 20 mM Tris, 200 mM NaCl, pH7.5. The resulting supernatants were loaded onto a Ni-NTA column and the column was washed with 10 column volumes of buffer containing 20 mM Tris, 200 mM NaCl, and 20 mM imidazole, pH 7.5. The bound SIRT2 was eluted with 20 mM Tris, 200 mM NaCl, and 250 mM imidazole, pH 7.5. The eluted protein fractions were cleavage by Ulp1 overnight, then loaded onto a Ni-NTA column and collected the flow out solution. The solution were incubated with 10 mM Myr Peptide (PKK(Tmy)TG) and 2 mM dithiothreitol for 1 h at 4°C and then further purified in 20 mM Tris, 50 mM NaCl, pH 7.5 buffer, using a Superdex G200 column (GE Healthcare). The peak corresponding to SIRT2 was pooled, concentrated to 10 mg/mL and stored at −80°C.

### SIRT2 activity assay

The activity of SIRT2 was assessed by high-performance liquid chromatography (HPLC). Purified SIRT2 was incubated in 60 μL of reaction buffer (20 mM Tris pH 8.0, 1 mM DTT, 1 mM NAD) with 32 μM H3K9, H2BK12, or H4K16 acyl peptides, respectively, at 37°C for indicated time. The reaction was quenched with 60 μL of 200 mM HCl and 320 mM acetic acid in methanol and spun down at 18,000 g for 10 min to remove the precipitated SIRT2. The supernatant was analyzed by HPLC on a Kinetex XB-C18 column (100 A, 75 mm × 4.6 mm, 2.6 μm, Phenomenex).

For kinetics, 0.2 μM SIRT2 was incubated in 60 μL of reaction buffer (20 mM Tris pH 8.0, 1 mM DTT, 1 mM NAD) with H3K9 acetyl or myristoyl peptides at varied concentrations at 37°C for 2 min (H3K9 acetyl) or 3 min (H3K9 myristoyl). Peptide concentration used for H3K9 acetyl was 1, 2, 4, 8, 16, 32, 64, 128 μM. Peptide concentration used for H3K9 myristoyl was 0.25, 0.5, 1, 2, 4, 8, 16, 32 μM. The reactions were quenched and analyzed as described above. The product and substrate peaks were quantified by integrating their absorbance area at 280 nm, which were converted to initial rates. The initial rates were then plotted against the peptide concentrations and fitted using the Kaleidagraph program.

### Crystallization of the SIRT2/BHJH-TM1

Crystals of SIRT2/BHJH-TM1 were grown using the vapor diffusion hanging drop method. A solution containing 10 mg/mL of SIRT2 in 20 mM Tris, 50 mM NaCl, pH 7.5 was preincubated on ice with 10 mM BHJH-TM1 for about 1 h. Hanging drops were formed by mixing 1.5 μL of protein solution and 1.5 μL of well solution containing 25% (v/v) PEG 3350, 0.1 M Hepes buffer, pH 7.5. Rod shape crystals grew in about 6 days to their maximum size of 0.2−0.3 mm × 0.1−0.2 mm.

### X-ray Data Collection and Processing

X-ray diffraction data for SIRT2/BHJH-TM1 were collected at Beamline A1 at the Cornell High Energy Synchrotron Source (CHESS) using an ADSC Quantum 210 CCD detector. The data collection temperature was 100 K. A total of 180° of data was collected with an oscillation range of 1° per frame and an exposure time of 5 s per frame. X-ray diffraction data were indexed, integrated, scaled, and merged using the program HKL2000^35^ to 2.1 Å resolution. Data collection and processing statistics are summarized in Table 2.

### Structure Determination and Refinement

The structure of SIRT2/BHJH-TM1 was determined by molecular replacement using Phaser^36^ as implemented in the *CCP4*^37^ program package. A monomer of human SIRT2 Apo-form (PDB ID: 3ZGO) was used as the search model. The initial model was refined to an R-factor of 28.74% and R-free of 37.71% by using the maximum likelihood method implemented in *REFMAC5* as part of *CCP4*^37^ program suite and was manually adjusted using COOT. After several cycles of refinement and model building, BHJH-TM1 and water molecules were added. The final model was refined to an R-factor of 22.1% and R-free of 27.3%. The Ramachandran plot shows 91.7% of residues in the most favorable regions, 8.1% in the allowed regions and 0.2% in generously allowed regions. No residue was in disallowed regions. Refinement statistics are summarized in Table 2.

### Data deposition

Atomic coordinates and structure factors for the reported crystal structure have been deposited with the Protein Data Bank under accession code 4R8M.

## Figures and Tables

**Table 1:** Kinetics data for SIRT2 on acetyl and myristoyl H3K9 peptides

Acyl peptide	*k*_cat_ (s^−1^)	*K*_m_ (μM)	*k*_cat_*/K*_m_ (s^−1^M^−1^)
H3K9 acetyl	0.275 ± 0.014	19.00 ± 0.85	14500
H3K9 myristoyl	0.018 ± 0.003	0.24 ± 0.03	74000

**Table 2:** Crystallographic data collection and refinement statistics of SIRT2/BHJH-TM1

Data collection	
Space group	*P2_1_*
Cell dimensions	
a, b, c (Å)	36.94, 116.80, 70.91
α, β, γ (°)	90.0, 91.8, 90.0
Resolution (Å)	50.00−2.10
Rsym or Rmerge (%)	10.5 (55.1)*
I/σI	9.92 (2.38)
Completeness (%)	97.7 (97.3)
Redundancy	4.5 (3.4)
**Refinement**	
Resolution (Å)	45.11−2.10
No. reflections	32677
Rwork/Rfree (%)	22.09/27.33
No. of protein residues	571
No. of ligand/ion molecules	
Myristoyl H3K9	2
Zn	2
No. of water	27
B-factors	46.72
r.m.s. deviations	
Bond lengths (Å)	0.009
Bond angles (°)	1.24

*Highest resolution shell is shown in parenthesis.
